# Development of a Cell-Based Reporter Potency Assay for Live Virus Vaccines

**DOI:** 10.3390/vaccines12070769

**Published:** 2024-07-13

**Authors:** Dengyun Sun, Brian K. Meyer, Dhanvanthri S. Deevi, Asra Mirza, Li He, Ashley Gruber, Susan J. Abbondanzo, Noah A. Benton, Melissa C. Whiteman, Robert C. Capen, Kevin B. Gurney

**Affiliations:** 1 Analytical Research & Development, Merck & Co., Inc., Rahway, NJ 07065, USAmelissa.whiteman@merck.com (M.C.W.); kevin_gurney@merck.com (K.B.G.); 2 Regulated Bioanalytical Immunogenicity & Molecular, Preclinical Development, Merck & Co., Inc., Rahway, NJ 07065, USA; 3 Research CMC Statistics, Merck & Co., Inc., Rahway, NJ 07065, USA

**Keywords:** vaccine potency assay, qualification, robustness, DoE

## Abstract

The rapid development of potency assays is critical in the development of life-saving vaccines. The traditional plaque assay or fifty percent tissue culture infectious dose (TCID_50_) assay used to measure the potency of live virus vaccines is time consuming, labor intensive, low throughput and with high variability. Described here is the development and qualification of a cell-based reporter potency assay for two vaccines for respiratory viral infection, one based on the recombinant vesicular stomatitis virus (rVSV) backbone, termed Vaccine 1 in this paper, and the other based on the measles virus vector, termed Vaccine 2. The reporter potency assay used a Vero E6 cell line engineered to constitutively express NanuLuc^®^ luciferase, termed the VeroE6-NLuc or JM-1 cell line. Infection of JM-1 cells by a live virus, such as rVSV or measles virus, causes a cytopathic effect (CPE) and release of NanuLuc^®^ from the cytoplasm into the supernatant, the amount of which reflects the intensity of the viral infection. The relative potency was calculated by comparison to a reference standard using parallel line analysis (PLA) in a log–log linear model. The reporter assay demonstrated good linearity, accuracy, and precision, and is therefore suitable for a vaccine potency assay. Further evaluation of the Vaccine 1 reporter assay demonstrated the robustness to a range of deliberate variation of the selected assay parameters and correlation with the plaque assay. In conclusion, we have demonstrated that the reporter assay using the JM-1 cell line could be used as a potency assay to support the manufacturing and release of multiple live virus vaccines.

## 1. Introduction

The COVID-19 pandemic has catalyzed the rapid development of life-saving vaccines using a variety of technologies [1,2]. Potency assays are a regulatory requirement for the release and stability of vaccine lots. Assessment of vaccine potency can be determined using in vitro, in vivo, or multiplex methods [1,2]. For live virus vaccines, infectivity assays such as the plaque assay or fifty percent tissue culture infectious dose (TCID_50_) assay are commonly used as the potency assay [3]. The plaque assay is considered the “gold standard” for measuring infectious virus titer [4,5,6]. The plaque assay is typically performed using 6- to 24-well plates and it can take 2–14 days for the plaque to form. The virus titer is reported as plaque-forming units per milliliter (PFU/mL). Variations of the plaque assay using fluorescently labeled antibodies specific for a viral antigen have also been developed and the titer is reported as focus-forming units per milliliter (FFU/mL) [7,8,9]. The TCID_50_ assay is another commonly used virological method to measure the infectious virus titer [10,11]. This endpoint dilution assay quantifies the amount of infectious virus required to produce a cytopathic effect (CPE) in 50% of inoculated cells using the Spearman–Karber or Reed–Muench methods. Since TCID_50_ is based on statistical estimation, typically 8–12 replicates per virus dilution are needed and a full 96-well plate is required to test 1 sample. Similarly, fluorescent antibodies can be used to visualize the CPE and shorten the assay time. The titer is typically reported as 50% fluorescent antibody infectious dose per milliliter (FAID_50_/mL) [12]. Molecular methods such as quantitative polymerase chain reaction (qPCR) that allows quantification of viral particles at the genome level have been evaluated for several viruses [13,14]. A reverse-transcription qPCR (RT-qPCR) method was developed to measure the potency of each component in the pentavalent rotavirus vaccine, RotaTeq^®^ [14]. The RT-qPCR method is fast, sensitive, specific, accurate, precise, and high throughput. On the other hand, the RT-qPCR requires multiple critical reagents including specific primes/probes and PCR mixtures, multiple assay steps, and long hands-on time.

In this study, we describe a NanuLuc^®^ reporter assay to measure the potency of two vaccine candidates for a respiratory viral infection. Vaccine 1 is based on the recombinant vesicular stomatitis virus (rVSV) backbone with the respiratory virus glycoprotein S replacing the VSV-G protein. Vaccine 2 is based on the measles virus vector with the same glycoprotein S inserted into the measles virus genome. Both vaccines are replication competent viruses causing cytopathic effect (CPE) similar to the parental virus, VSV and measles virus, respectively. The reporter assay uses a Vero E6 cell engineered to express NanuLuc^®^ luciferase under a cytomegalovirus (CMV) promoter (named VeroE6-NLuc or JM-1 cell line). The JM-1 cell line was previously evaluated to measure the potency of the VSV-based Ebola vaccine, ERVEBO^®^ [15]. Therefore, it was hypothesized that this cell line could also be used to evaluate Vaccine 1 and Vaccine 2 potency. The Vaccine 1 reporter assay using the JM-1 cell line demonstrated acceptable linearity, accuracy, precision, robustness, and showed a good correlation with the plaque assay. The same reporter assay was adapted to Vaccine 2 with similar or better assay performance. These results indicate that the reporter assay using the JM-1 cell line has broad application in determining the potency of multiple live virus vaccines as shown here for representative viruses from the *Rhabdoviridae* and *Paramyxoviradae* families.

## 2. Materials and Methods

### 2.1. Cells and Viruses

The JM-1 cell line (VeroE6-NLuc) was generated by engineering a CMV promoter to drive constitutive NanuLuc^®^ Luciferase expression in VeroE6 cells as described previously [15]. Briefly, A DNA fragment consisting of a CMV promoter was fused to the NanuLuc^®^ gene and cloned into pGL4.17 vector (Promega, Madison, WI, USA). VeroE6 (ATCC, Manassas, VA, USA) cells were transfected with the pGL4.17-CMV-NanuLuc^®^ construct using Lipofectamine 2000 (Invitrogen, Waltham, MA, USA). G418 resistant clones were selected and screened for NanuLuc^®^ expression. The final single clone, termed JM-1, was expanded and cryopreserved for future use. The JM-1 cells were cultured in growth medium containing MEM (Gibco, Waltham, MA, USA) supplemented with 10% heat inactivated fetal bovine serum (FBS, Hyclone, Logan, UT, USA), 100 U/mL of penicillin-streptomycin (Gibco), 0.5 mg/mL G418 (Gibco), and maintained in a humidified 37 ± 1 °C, 5 ± 1% CO_2_ incubator. The JM-1 cell infection medium contained 2% FBS and the same antibiotics as indicated above. All Vaccine 1 and 2 materials were manufactured at Merck & Co., Inc., West Point, PA, USA. The titer of the Vaccine 1 reference standard was determined by the plaque assay and the titer of Vaccine 2 reference standard was determined by TCID_50_.

### 2.2. Reporter Assay

**Figure 1:**
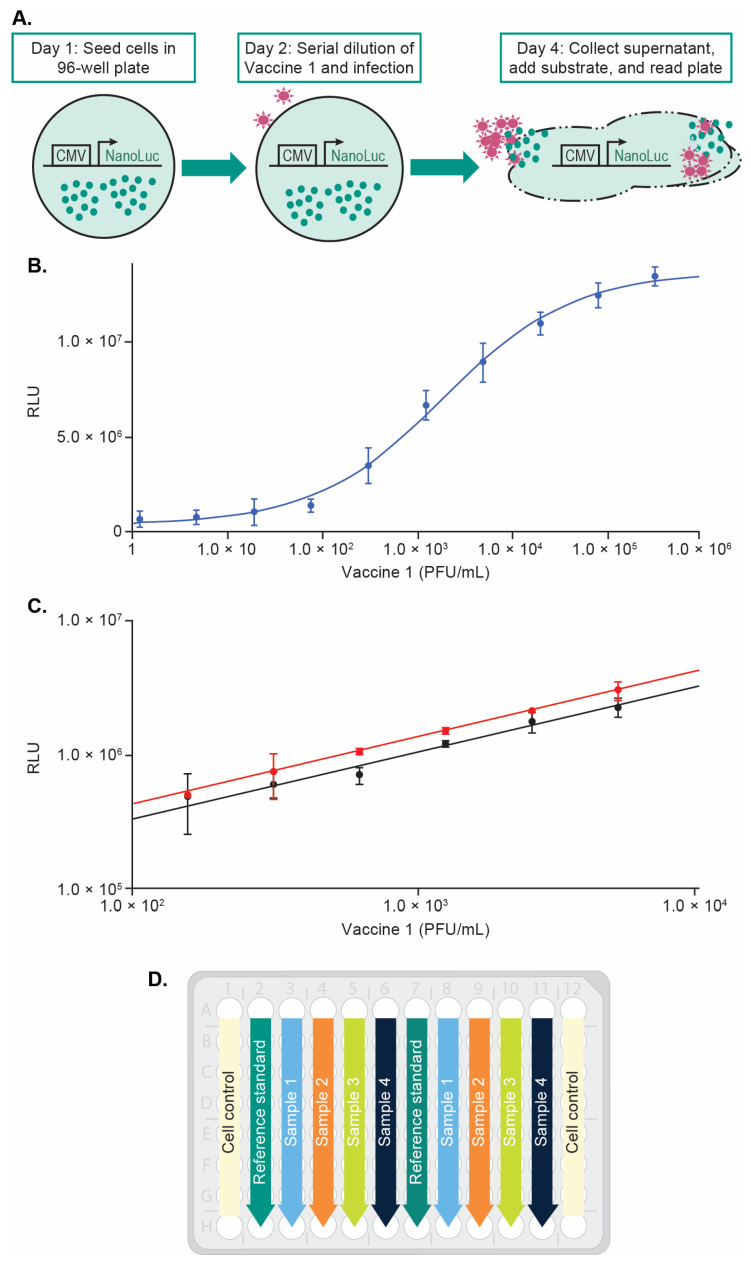
Vaccine 1 reporter assay. (**A**) The principle of the Vaccine 1 reporter assay. (**B**) Vaccine 1 dose response curve at 48 h. Data were fit using a 4-PL model in Softmax Pro 7.0. (**C**) Log–log linear model and the relative potency calculation. Reference standard (red line) and a test sample (black line) were fit globally (common slope) using log–log in Softmax Pro 7.0. The error bars represent the standard deviation for duplicated RLU. (**D**) Plate map for the Vaccine 1 reporter assay. Up to 4 samples in duplicate can be tested together with the reference standard on 1 plate.

The principle of the Vaccine 1 reporter assay is described in Figure 1A. Infection of JM-1 (VeroE6-NLuc) cells by Vaccine 1 causes CPE and release of NanuLuc^®^ from the cytoplasm into the supernatant. The supernatant is collected and incubated with the NanuLuc^®^ substrate, and the resulting luminescence is measured in a plate reader. In the final version of the Vaccine 1 reporter assay, the JM-1 cells were seeded in 96-well plates at 1.0 × 10^4^ cells per well in 100 µL growth medium on Day 1. On Day 2, the Vaccine 1 reference standard was pre-diluted to 2.0 × 10^4^ PFU/mL in duplicate and then serially diluted 2-fold in infection medium. Testing samples with unknown titers will be diluted the same as the reference standard. Based on the estimated titer range, the pre-dilution of testing samples could be different from the reference standard, and the potency calculation will be adjusted accordingly. The culture medium of JM-1 cells in the 96-well plates was removed and the cell monolayer was infected by the serially diluted reference standard and testing samples as described in Figure 1D. After 2 days post-infection, the supernatants from the 96-well plates were collected and mixed with Nano-Glo^®^ substrate (Promega) per the manufacturer’s instructions. The luminescence was measured using a SpectraMax L (Molecular Devices) and the mean of relative luminescence units (RLUs) were fit against the Vaccine 1 concentration (PFU/mL) using a log–log model in SoftMax Pro 7.0. The percentage relative potency (%RP) of the test sample was determined by comparing it to a concurrently analyzed reference standard using parallel line analysis (PLA) [16]. The relative potency of the Vaccine 1 test sample is calculated as the geometric mean of three relative potency determinations obtained from three independently handled assay plates. The Vaccine 2 reporter assay using the JM-1 cell line was developed similar to Vaccine 1 with the same assay principle. To generate the optimal signal and improve the assay performance, the starting concentration of the Vaccine 2 reference standard was optimized to 2.5 × 10^4^ TCID_50_/mL and the luminescence was measured after 4 days post-infection.

### 2.3. Vaccine 1 Reporter Assay Qualification

To assess the Vaccine 1 reporter assay performance, five samples corresponding to the target RP% levels of 400%, 200%, 100%, 50%, and 25% of the reference standard were tested by two analysts on two separate days with two runs per day per analyst (n = 8). A linear regression analysis was performed using the measured RP% as the dependent variable and the target RP% as the independent variable on a log scale. Linearity was assessed by the %dilution bias, which is defined as %dilution bias = $100\times (2^{b-1}-1)$, where b is the estimated slope from the linear regression. Accuracy was assessed based on the %relative bias for each %RP level which is calculated according to the formula: %relative bias = 100 × (Geomean/Target RP% − 1), where the geomean refers to the geometric mean of all the RP% results corresponding to a particular target RP% level. Precision measures the overall variability under a variety of normal testing conditions within one laboratory. It includes repeatability (within-run variability) and intermediate precision (sum of within-run and between-run variability). A random effect model was assumed to estimate the variance components due to the analyst, day, and residual. The repeatability percent relative standard deviation (%RSD) was calculated using the following equation: $Repeatability\;\%RSD=100\times \sqrt{e^{{\hat{\sigma }}_{intra}^{2}}-1}\;;$ and the intermediate precision %RSD was calculated using the following equation: $Intermediate\;Precision\;\%RSD=100\times \sqrt{e^{{\hat{\sigma }}_{intra}^{2}+{\hat{\sigma }}_{inter}^{2}}-1}$, where ${\hat{\sigma }}_{intra}^{2}$ refers to within-run variability, and ${\hat{\sigma }}_{inter}^{2}$ refers to between-run variability.

### 2.4. Vaccine 1 Reporter Assay Robustness

The robustness of the Vaccine 1 reporter assay was evaluated by testing a deliberately varied range of selected assay parameters. In the robustness study, the Vaccine 1 drug substance, formulated at an estimated titer of 2.0 × 10^6^ PFU/mL, was used as the reference standard and for preparation of the test samples (25%, 50%, 100% and 200% RP). The critical assay parameters selected for the robustness study included cell seeding density (cells per well), Vaccine 1 starting concentration (PFU/mL), JM-1 cell seeding time (hours), infection time (hours) cell passage (low, high), and substrate incubation time (minutes) as shown in Table 1. A D-optimal design for the five parameters (excluding substrate incubation time) with eight random blocks (runs) was used, with two analysts each performing four runs on different days. Altogether there were 32 plates including 8 center points, with each plate read at 10 min and then 20 min (substrate incubation time). The assay performance including linearity, accuracy, and precision was evaluated using R 4.1.0 software. The effects of the factors on %RP (main effect and two-way interaction) were assessed using JMP^®^ version 15.2 (SAS Institute).

### 2.5. Vaccine 1 Reporter Assay and Plaque Assay Correlation

To compare the reporter assay to the gold standard plaque assay, twelve samples from Vaccine 1 process development and forced degradation studies with a wide titer range of 2.9 × 10^4^ to 1.0 × 10^8^ PFU/mL by the plaque assay were tested in the Vaccine 1 reporter assay. For the Vaccine 1 plaque assay, Vero cells (ATCC) were seeded in complete growth medium containing DMEM/high glucose (Gibco), 10% heat inactivated FBS, and 100 U/mL penicillin-streptomycin in 24-well plates. The next day, Vaccine 1 was serially diluted with assay medium (DMEM/high glucose + 2% FBS), and 4 dilution points in singlet were inoculated onto a Vero cell monolayer. After 60 min absorption, the freshly made and pre-warmed overlay medium (complete growth medium + 0.5% Agarose) was overlaid on the Vero cell monolayer. After 2 days post-infection, the cells were fixed with a formaldehyde solution (Sigma-Aldrich, St. Louis, MO, USA) and stained with a 1% crystal violet solution (Sigma-Aldrich). The Vaccine 1 titer (PFU/mL) was calculated based on all dilutions where the geometric mean of observed plaques ranged from 5 to 70. To assess the correlation between the plaque assay and the reporter assay, the plaque titers (PFU/mL) were converted into %RP by comparison to the reference standard (100 × sample titer/reference titer). For the Vaccine 1 reporter assay, the reference standard and selected samples were pre-diluted to 1.0 × 10^4^ PFU/mL based on the known plaque titers and tested by one analyst with three independent plates on the same day. The %RP of a test sample is reported as the geometric mean of three relative potency determinations from the PLA, multiplied by the pre-dilution factor of the sample and divided by the pre-dilution factor of the reference standard.

## 3. Results

### 3.1. Vaccine 1 Reporter Assay Development

To prove the concept of the Vaccine 1 reporter assay, JM-1 cell monolayers were infected with Vaccine 1 for 1–3 days and the supernatant was collected and mixed with Nano-Glo^®^ (Promega) for luminescence measurement. The RLU was plotted against the Vaccine 1 concentration using a four-parameter logistic (4-PL) model. The luminescence measured at 2 days post-infection generated the best dose response curve and was shown in Figure 1B. To further optimize the assay, cell seeding density, Vaccine 1 infection time, FBS concentration in the infection medium, different plate readers were evaluated. To increase the assay sensitivity and throughput, a log–log linear model instead of 4-PL was used to calculate the RP% in the final method. Figure 1C shows the global fitting of a test sample with a target RP% at 50% using a log–log model. By using a log–log model which only needs six dilution points (2-fold dilution) per sample, up to four samples together with a reference standard in duplicate can be tested per plate without using row A and H (Figure 1D).

### 3.2. Vaccine 1 Reporter Assay Qualification

**Figure 2:**
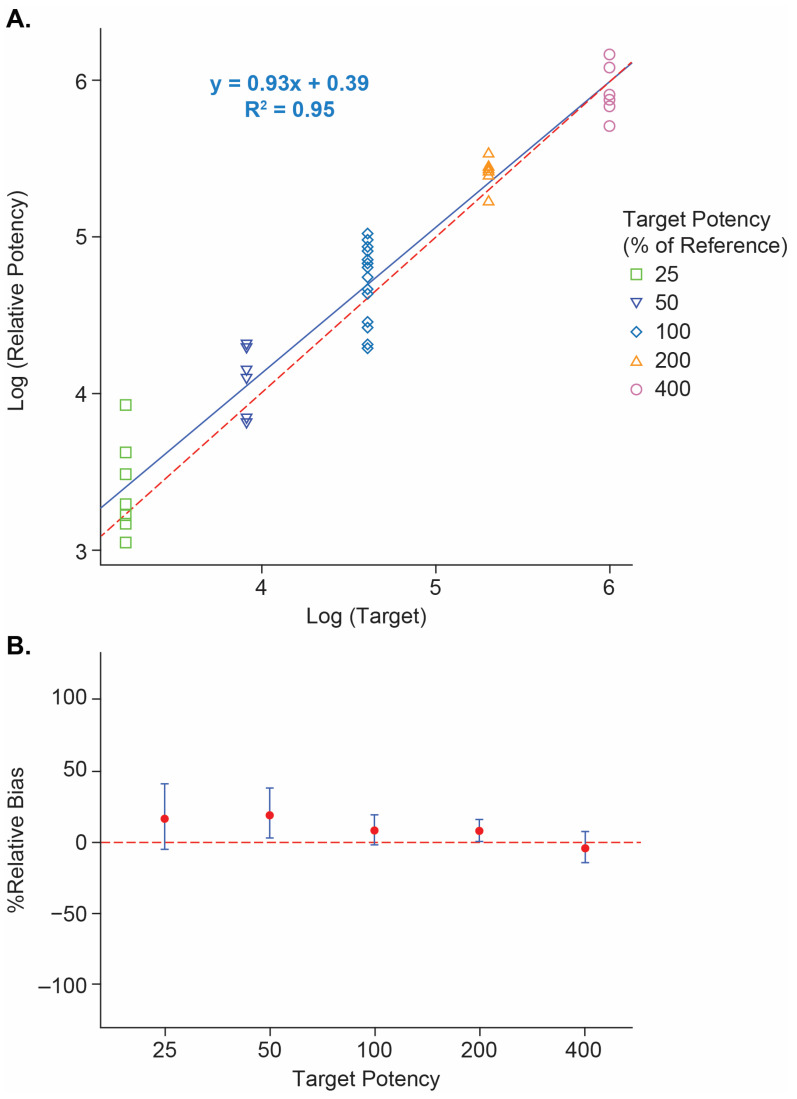
Linearity from the Vaccine 1 reporter assay pre-qualification. (**A**): Linearity of the Vaccine 1 reporter assay in the range of 25–400% relative potency on a log scale. X-axis: target RP% on log scale. Y-axis: measured RP% on log scale. Blue solid line: fitted line. Red dashed line: y = x. (**B**): %relative bias of Vaccine 1 reporter assay. Red dot: relative bias% estimates. Error bar: 90% CI. Red dashed line: y = 0.

The Vaccine 1 reporter assay was qualified to assess the assay performance including dilutional linearity, accuracy, and precision (repeatability and intermediate precision). The Vaccine 1 reporter assay dilutional linearity was shown in Figure 2A with a coefficient of determination *R*^2^ = 0.95 and slope = 0.94 in the testing range of 25% to 400% relative potency. The overall %dilution bias is estimated as −4.6% (Table 2). The %relative bias estimates at each target RP% level with their corresponding 90% confidence intervals (CI) were plotted in Figure 2B. The largest %relative bias is 19.0% at 50% RP level with 90% CI (3.0%, 37.5%) (Table 2). The overall repeatability %RSD was 16.8% and the intermediate precision %RSD was 25.2% based on all samples. The assay performance from the qualification study was summarized in Table 2.

### 3.3. Vaccine 1 Reporter Assay Robustness Study

**Figure 3:**
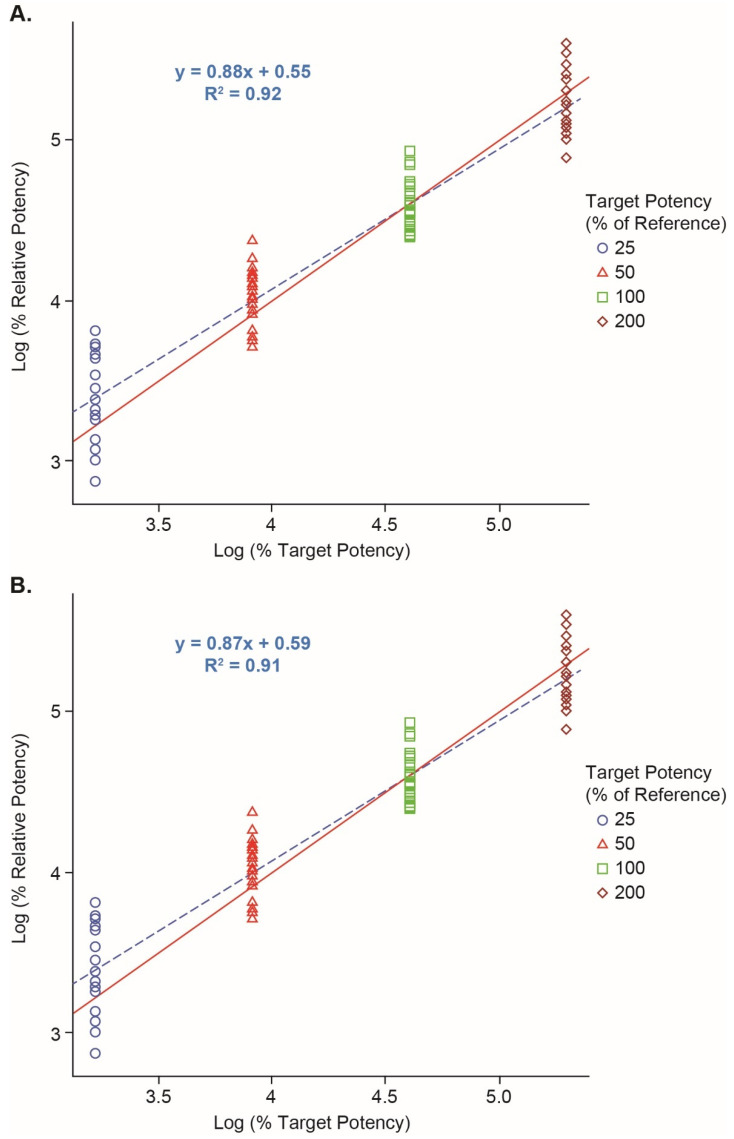
Linearity from the Vaccine 1 reporter assay robustness study. (**A**) Linearity of Log(RP%) results (25–200%) read at 10 min of substrate incubation time (n = 32). (**B**) Linearity of Log(RP%) results read at 20 min (n = 25 due to missing data). Red solid line: y = x. Blue dashed line: fitted line.

Both the assay performance and the effects of the assay parameters were evaluated in the robustness study. Overall, the results showed acceptable performance of the assay in terms of dilutional linearity, accuracy, and precision over the range of 25% to 200% relative potency. Figure 3A showed the dilutional linearity plot for the data at 10 min substrate incubation. The linearity was demonstrated by the fitted line y = 0.88x + 0.55 (*R*^2^ = 0.92), where y is the log (measured RP%), and x is the log (target RP%). For accuracy, the largest %relative bias was 16.6% with the target at 25% for 10 min substrate incubation (Table 3). For precision, the overall repeatability %RSD and intermediate precision %RSD based on all samples were 20.4% and 20.7%, respectively, for 10 min substate incubation (Table 3). There was no significant main effect for each of the four target RP% levels, suggesting that the assay was robust to the evaluated parameters. Similar results were found for 20 min of substrate incubation and the data are shown in Figure 3B and Table 3. The results indicate that the Vaccine 1 reporter assay was robust to the assay parameters evaluated here and suitable for its intended purposes.

### 3.4. Correlation between the Reporter Assay and Plaque Assay

**Figure 4:**
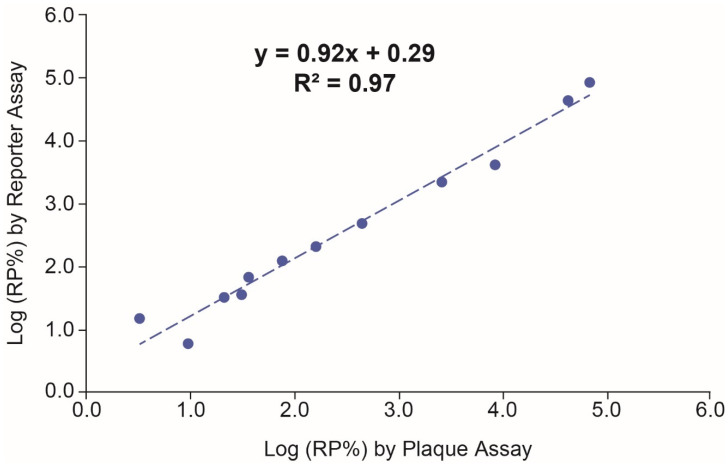
Correlation between the Vaccine 1 reporter assay and the plaque assay. The geomean of RP% from the reporter assay (n = 3) was fitted against the geomean of RP% from plaque assay on log scale (n = 6). The blue dashed line is the fitted line. The linear regression equation is shown in the graph.

The correlation between the Vaccine 1 reporter assay and the plaque assay was evaluated by testing twelve samples with a wide range of plaque titers. As shown in Figure 4, the Log(RP%) from the reporter assay had good linear regression with Log(RP%) from the plaque assay (y = 0.92x + 0.29, *R*^2^ = 0.97), suggesting that the Vaccine 1 reporter assay had good correlation with the plaque assay. The RSD% of Vaccine 1 reporter assay from the twelve samples were all ≤29% (n = 3), which in general were smaller than those in the plaque assay (highest RSD% = 45.9 with n = 6), showing that the Vaccine 1 reporter assay has better precision than the plaque assay.

### 3.5. Vaccine 2 Reporter Assay

**Figure 5:**
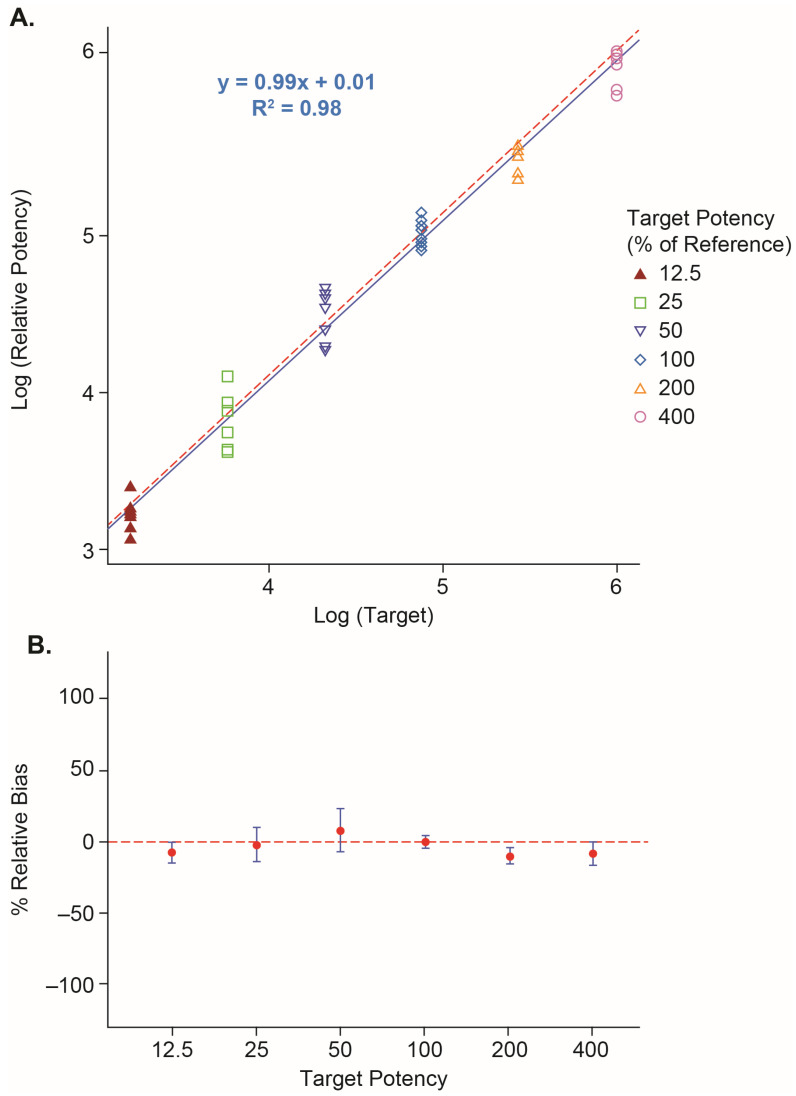
Linearity of the Vaccine 2 reporter assay. (**A**) Linearity of the Vaccine 2 reporter assay in the range of 12.5–400% RP levels. X-axis: target RP% on log scale. Y-axis: measured RP% on log scale. Blue solid line: fitted line. Red dashed line: y = x. (**B**) %relative bias of Vaccine 2 reporter assay. Red dot: relative bias estimates. Error bar: 90% CI. Red dashed line: y = 0.

The Vaccine 1 reporter assay using the JM-1 cell line was also evaluated for Vaccine 2 with minor changes of assay parameters including starting concentration and infection time. To assess the performance of the Vaccine 2 reporter assay, dilutional linearity, accuracy, and precision were estimated similarly. Six constructed samples at RP% levels from 12.5% to 400% were tested by two analysts on two days with two runs per analyst per day (n = 8). As shown in Figure 5A, The Vaccine 2 reporter assay had good dilutional linearity with *R*^2^ = 0.98 and slope = 0.99. The overall %dilution bias based on all samples was estimated to be −0.8%. The % relative bias of each target RP% level and the corresponding 90% CI are shown in Figure 5B. The largest %relative bias was −10.6% at the 200% RP level with 90% CI (−16.0, −4.8). The overall repeatability %RSD was 14.0% and the intermediate precision %RSD was 16.0% based on all samples tested (Table 4), suggesting good assay performance.

## 4. Discussion

Reporter assays using luminescence are widely used to study gene regulation, signal transduction, molecular mechanisms, drug screening, and biologics potency assays [17,18,19,20,21]. NanuLuc^®^ luciferase (often with PEST destabilization domain), developed by Promega, offers several advantages over firefly and Renilla luciferases, including smaller size, brighter luminescence, lower background, and enhanced signal stability [19]. However, there are few applications of reporter assays in the vaccine field so far. In this study, we have developed a novel NanuLuc^®^ reporter assay to measure the potency of two live virus vaccine candidates for a respiratory viral infection. Since the principle of the reporter potency assay is based on the NanuLuc^®^ secretion because of the cytopathic effect, which is very common for live virus infections, we believe it has broad application for many live viruses, which will be tested in future studies.

While the 4-PL model is widely used in biological assays [17,20,21], in this study we chose a log–log model for the Vaccine 1 and Vaccine 2 reporter assays, which increased the assay sensitivity and testing throughput. Using a 4-PL model, a high starting titer (concentration) of Vaccine 1, estimated at 1.0 × 10^6^ PFU/mL or higher, was needed to reach the upper asymptote. This is challenging for testing Vaccine 1 samples with low titers such as forced degradation samples. For Vaccine 1 reporter assays using the log–log model, the starting concentration (typically below the estimated EC_50_) was set at 2.0 × 10^4^ PFU/mL, which increased the assay sensitivity by at least 50-fold. In addition, because only six points (or less) are needed to fit a line in the log–log model compared to 11 points in the 4-PL model, the assay using a log–log linear model is much simpler and allows for an increased number of samples tested per plate.

The Vaccine 1 reporter assay has demonstrated good dilutional linearity, precision, accuracy, and robustness. In addition, the relative potency from the Vaccine 1 reporter assay correlated well with that from the plaque assay. Compared to the Vaccine 1 plaque assay which requires fixation-staining steps and has an overall intermediate precision 31.0% RSD, the Vaccine 1 reporter assay is much simpler and has better intermediate precision (25.2%RSD or less). For Vaccine 2, the current release method TCID_50_ takes 7–8 days, requires one 96-well plate to test one sample, and has intermediate precision 38.3%RSD. Our Vaccine 2 reporter assay has faster turnaround time (5 days), higher throughput (4 samples per plate) and much better intermediate precision (16.0%RSD). The lower assay variability, faster turnaround time, higher throughput and easier operation of the reporter potency assay using the JM-1 cell line make it a better alternative to the traditional plaque assay or TCID_50_ for release and stability testing of vaccines.

Despite the advantages of the reporter potency assay as described in this study, one limitation of the reporter assay is that it cannot directly measure the viral titer as plaque assay or TCID_50_. The potency of a testing sample in the reporter assay is calculated as the relative potency by comparing it to the concurrently analyzed reference standard. The titer of a testing sample can then be calculated using the relative potency and the known titer of the reference standard. In the early vaccine development stage when a reference standard is not available yet, it might be challenging to use the reporter potency assay for the release and stability testing of vaccine lots. Once a reference standard is established and the viral titer is known, the reporter assay shows big advantages in terms of assay variability, turnround time, operational ease, and testing throughput.

## 5. Conclusions

A cell-based reporter assay was successfully developed as an alternative potency assay for live virus vaccines. The reporter potency assay has demonstrated good assay performance, robustness to selected assay parameters, and correlation with the plaque assay.

## Figures and Tables

**Table 1:** Applied variation in the Vaccine 1 reporter assay robustness DOE study.

Parameter	Low	Set Point		High
Cell seeding density (cell/well)	8000	10,000		12,000
Cell seeding time (hours)	20	24		26
Reference concentration (PFU/mL)	8000	10,000		12,000
Infection time (hours)	44	46		48
Cell passage	Low (≤25)		High (≥30)	
Substrate incubation time (min)	10		20	

**Table 2:** Summary of Vaccine 1 reporter assay qualification.

Performance Characteristics	Results
Dilutional Linearity	The slope, $R^{2}$ and root mean square error (RMSE) of the regression analysis based on all 8 runs are estimated to be 0.94, 0.95 and 0.21, respectively.The %dilution bias based on the overall regression analysis is calculated to be −4.6%.
Accuracy	The largest %relative bias is 19.0% at the 50% potency level with the 90% CI being (3.0%, 37.5%).
Repeatability	The overall %RSD for repeatability is 16.8%.
Intermediate Precision	The overall %RSD for intermediate precision is 25.2%.

**Table 3:** Summary of Vaccine 1 reporter assay robustness study.

Substrate Incubation Time	Target RP%	n	Geomean	%Relative Bias	Repeatability	Intermediate Precision
10 min	25	32	29.2	16.6	29.4	29.4
	50	32	54.9	9.8	16.8	17.3
	100	32	98.7	−1.3	15.1	15.3
	200	32	183.0	−8.5	17.3	17.9
	Overall				20.4	20.7
20 min	25	25	29.3	17.1	30.9	31.9
	50	25	56.0	11.9	16.4	17.3
	100	25	99.6	−0.4	14.7	15.1
	200	25	180.9	−9.5	18.4	19.1
	Overall				21.0	21.9

**Table 4:** Summary of Vaccine 2 reporter assay qualification.

Performance Characteristics	Results
Dilutional Linearity	The slope, $R^{2}$ and RMSE for the overall regression analysis are estimated to be 0.99, 0.98 and 0.14, respectively. The %dilution bias based on the overall regression analysis is calculated to be −0.8%.
Accuracy	The largest %relative bias is −10.6% at the 200% potency level with the 90% CI being (−16.0%, −4.8%).
Repeatability	The repeatability %RSD based on all samples is 14.0%.
Intermediate Precision	The intermediate precision %RSD based on all samples is 16.0%.

## Data Availability

The data presented in this study are available in this article.
